# Phytochemical Composition and Characterization of In Vitro Bioactivities from *Pinus* Using Green Process

**DOI:** 10.3390/molecules29225295

**Published:** 2024-11-09

**Authors:** Amel Chammam, Mehrez Romdhane, Luc Fillaudeau, Jalloul Bouajila

**Affiliations:** 1 Energy, Water, Environment, and Process Laboratory, (LR18ES35) National Engineering School of Gabes, University of Gabes, Gabes 6029, Tunisia; chammam@insa-toulouse.fr (A.C.); mehrez.romdhane@univgb.tn (M.R.); 2 Toulouse Biotechnology Institute, Bio & Chemical Engineering TBI (CNRS UMR5504, INRAE UMR792, INSA Toulouse), 31400 Toulouse, France; luc.fillaudeau@insa-toulouse.fr; 3 Laboratoire de Génie Chimique, Université de Toulouse, CNRS, INP, UPS, F-31062, 31400 Toulouse, France

**Keywords:** bioactive compounds, pinecone extracts, antioxidant activity, extraction, component analysis, chromatography analysis

## Abstract

*Pinus* species are notable in Mediterranean regions due to their ecological and economic importance. Various parts of these species are widely used in traditional medicine, especially pinecones, which are a significant source of bioactive compounds. The current study aimed to evaluate the phytochemical composition and biological properties of the aqueous extracts obtained by maceration from three *Pinus* petal fractions, from *P. halepensis* Mill., *P. brutia* Ten., and *P. pinea* L. (APW, BPW, and PPW respectively), and the core fractions of the same species (ACW, BCW, and PCW respectively). The results showed that APW demonstrated superior performance compared to other species and fractions (*p* ≤ 0.05), with the highest total polyphenol content (203.51 mg GAE/g DW) and the highest antioxidant potential (IC_50_ = 13.51 µg/mL) against DPPH free radical. All extracts showed high anticancer activity against HeLa and HepG2 cancer cell lines, and low inhibition against HEK-293, a normal cell line (<15%), indicating that none of extracts have any toxicity effect. Furthermore, only APW exhibits a significant inhibition against α-glucosidase with 77.20% at 50 µg/mL. HPLC-DAD analysis was conducted to identify 14 compounds. GC-MS analysis was conducted to identify 28 compounds, of which 11 were detected for the first time in this species. This study offers valuable insights into phytochemistry and potential therapeutic applications of pinecones.

## 1. Introduction

The *Pinus* genus belongs to the *Pinaceae* family, within which there are 250 species, including *Aleppo* (*P. halepensis* Mill.), *Brutia* (*P. brutia Ten.*), and *Pinea* (*P. pinea* L.) pines [1]. These species grow in the northern hemisphere, especially in the Mediterranean region, Asia, Europe, and North and Central America, known for their antioxidant, anti-inflammatory, anticancer, antifungal, and anti-microbial activities [2].

Pinecones from these species have been considered low-value byproducts, often used as firewood, discarded, or left to decompose, resulting in wasted biological resources and environmental pollution. However, studies have shown that these pinecones are rich in bioactive compounds such as proteins, polysaccharides, and polyphenols, which offer health benefits [3,4,5]. They also possess high antioxidant, antibacterial, and anti-inflammatory properties, and can inhibit the growth of cancer cells [6].

Many advanced techniques, such as microwave-assisted extraction (MAE) and ultrasound-assisted extraction (UAE), are increasingly used to extract bioactive compounds. These methods offer several advantages over traditional techniques, including reduced extraction time, lower solvent usage, minimal environmental impact, and high efficiency [7]. In addition, alternative techniques like accelerated solvent extraction (ASE) and supercritical fluid extraction (SFE) are gaining attention due to their ability to operate under higher pressure and temperature conditions, enhancing extraction yields and selectivity [8,9]. Despite these advancements, traditional methods such as Soxhlet extraction and maceration remain valuable. Maceration, in particular, stands out for its simplicity, low cost, minimal equipment needs, and environmental friendliness. Its efficiency, however, depends on factors such as agitation speed and duration, which influence mass transfer rates and overall extraction effectiveness [10].

Various analytical techniques are employed to identify the chemical composition of plant extracts, including gas chromatography (GC) and high-performance liquid chromatography (HPLC) [11,12,13]. GC is particularly effective for analyzing volatile compounds due to its high sensitivity and selectivity. To detect polar and less volatile compounds with GC analysis, derivatization methods are often used to enhance volatility and reduce interactions with the analytical system, including trimethylsilyl (TMS) derivatization, as well as aldononitrile acetate and alditol acetate methods [14]. Among these, TMS derivatization is the most widely used due to its ability to significantly increase compound volatility and improve thermal stability compared to other derivatization approaches [15].

According to our previous research, *P. halepensis* Mill., *P. brutia* Ten., and *P. pinea* L. pines, growing in Mediterranean regions, hold ecological and economic importance and would produce and accumulate more secondary metabolites, such as polyphenols, flavonoids, anthocyanins, and sterols. Based on the valorization of byproducts and environmental protection, the isolation of bioactive compounds from pinecones of these species possesses a bright prospect using green and natural solvents, such as water. To optimize extraction and better understand pinecone benefits, petals and core fractions were studied separately, as they may contain distinct bioactive compounds, enabling more precise and efficient extraction. Additionally, knowing the characteristics of the bioactive compounds extracted is very important when improving bioavailability.

This study aimed to extract bioactive compounds from the petal fractions of three pine species, *Aleppo* (*P. halepensis* Mill.), *Brutia (P. brutia* Ten.), and *Pinea (P. pinea* L.) (APW, BPW, and PPW), and their core fractions (ACW, BCW, and PCW) using aqueous maceration. The extracts were analyzed using high-performance liquid chromatography (HPLC) and gas chromatography–mass spectrometry (GC-MS), and their total phenolic content (TPC) and reducing sugar content (RSC) were measured. Additionally, the extracts were tested in vitro for antioxidant, anticancer, and antidiabetic activities.

To our knowledge, this is the first study to employ aqueous extracts obtained by maceration technique to explore chemical compositions and in vitro bioactivities from both petals and core fractions of these pine species. This innovative approach not only reveals the potential of underutilized pinecone fractions but also opens new avenues for their applications in health and nutrition.

## 2. Results and Discussion

### 2.1. Moisture Content and Particle Size

The pinecones utilized were naturally sun-dried on the forest floor, eliminating the need for an artificial drying process. Therefore, after dividing into petals and cores and grinding materials, the moisture content and the particle size were measured. 

The relative water content (n), absolute water content (x), and mean diameter of the materials from the petal and core fractions of three pine species are reported in Table 1.

Statistical analysis revealed no significant differences between species or fractions analyzed on moisture content and particle size. The average relative water content was approximately 10% (g water/g hm), while the absolute water content was around 9% (g water/g dm), consistent with their hygroscopic structure. These low values enable the cones to respond effectively to changes in ambient humidity, promoting the release and dispersal of seeds. The average particle size of samples was approximately 90 µm.

### 2.2. Extraction Yields

The extraction yields for the six extracts (APW, ACW, BPW, BCW, PPW, and PCW), obtained by aqueous maceration, were quantified and reported in Table 2. The solid extracts are characterized by a dark brown color and possess a woody, mild, and sweet odor.

Statistical analysis reveals significant variations in the yield values among the extracts based on the fractions and species used (*p* ≤ 0.05). For all extracts, petal extracts demonstrated higher yields compared to core extracts (*p* ≤ 0.05). The highest yields were reported for BPW extract (3.93%), followed by APW extract (2.47%), while PCW extract showed the lowest yield with 1.01%. The differences in yield between the pine species and parts highlight the importance of choosing the right species to optimize the extraction process.

Compared to the literature, no prior investigations had been conducted regarding the aqueous extraction by maceration for both petal and core fractions from *Pinus*. The study of Costa et al. [16] used ultrasound-assisted extraction with a 50/50 ethanol/water solution at a concentration of 100 g/L for 15 min at 50 °C to extract bioactive compounds from *Pinus pinea* petals and cores. They achieved higher extraction yields of 3% for petals and 2.4% for cores, which is better than the results of the current study. This difference may be due to the efficacity of ultrasound-assisted extraction. However, these findings confirm our hypothesis that the petal fraction had a higher yield (3%) than the core fraction (2.4%). 

This encourages us to continue our research, focusing on the chemical composition to understand the factors contributing to the petal fraction’s superior yield.

### 2.3. Chemical Compositions

The significantly different extraction yields between species, along with the superior yield of the petals fraction compared to the cores, have led to further investigation of their chemical composition. This aims to better understand the factors responsible for these variations.

#### 2.3.1. Reducing Sugar Content (RSC)

The RSC values acquired for the six extracts (APW, ACW, BPW, BCW, PPW, and PCW) are reported in Table 3. 

Statistical analysis reveals significant variations in the RSC values among the extracts based on the fractions and species used (*p* ≤ 0.05). For all extracts, petal extracts had higher RSC than core extracts, except *P. brutia* Ten. pine (*p* ≤ 0.05). The highest RSC was observed with BCW extract (470.09 mg GE/g DW), followed by the BPW extract (404.55 mg GE/g DW) and APW extract (325.12 mg/g DW) (*p* ≤ 0.05), while PCW extract showed the lowest concentration with 158.31 mg GE/g DW. These results illustrate the richness of pine species in reducing sugars.

Compared to the literature, the aqueous extract obtained after 2 h of maceration by Gamli [17] from *P. brutia* Ten. pinecone was reported to have a lower concentration of reducing sugars (approximately 227 mg GE/g DW) than that found in this study. In the current research, petal and core fractions were analyzed separately, and a maceration time of 24 h was used. This extended extraction time and the separation of fractions likely led to a higher extraction of reducing sugars, highlighting the effectiveness of this method in extracting bioactive compounds.

#### 2.3.2. Total Polyphenol Content (TPC)

**Figure 1:**
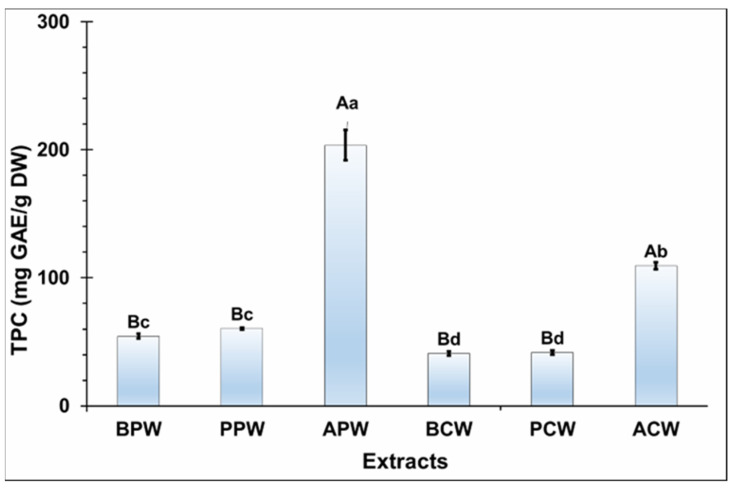
Total phenolic content (TPC). APW: *P. halepensis* Mill. petal water extract; ACW: *P. halepensis* Mill. core water extract; BPW: *P. brutia* Ten. petal water extract; BCW: *P. brutia* Ten. core water extract; PPW: *P. pinea* L. petal water extract; PCW: *P. pinea* L. core water extract; GAE: gallic acid; DW: dry weight. Each letter on the table represents a significant difference (*p* ≤ 0.05). Uppercase and lowercase letters mean a significant difference in species and fractions, respectively. Results are mean ± SD (n = 3).

The TPC of the six extracts (APW, ACW, BPW, BCW, PPW, and PCW) was determined and illustrated in Figure 1. 

Statistical analysis reveals significant variations in the TPC values among the extracts based on the fractions and species used (*p* ≤ 0.05). *P. halepensis* Mill. pine extracts had higher TPC than *P. brutia* Ten. and *P. pinea* L. pines extracts (*p* ≤ 0.05). For all extracts, petal extracts showed higher TPC than core extracts (*p* ≤ 0.05). The highest TPC values were observed in the APW extract (203.51 mg GAE/g DW), followed by the ACW extract (109.53 mg GAE/g DW), while the lowest TPC were observed in BCW and PCW extracts (42 mg GAE/g DW). 

The significant total phenolic content (TPC) values obtained from all extracts demonstrate that water is an effective solvent for extracting polyphenols. Its high polarity facilitates the inhibition of polyphenol oxidase activity, thereby preventing the oxidation of phenolic compounds [18].

Compared to the literature, the study of Belarbi [19] found that the TPC in the aqueous extract of *P. halepensis* Mill. pinecones was approximately 260 mg GAE/g DW, which falls within the range of our results. Furthermore, the study of Costa et al. [16] demonstrated that using ultrasound to extract phenolic compounds from *P. pinea* L. petals and cores resulted in higher TPC in the petals (601.8 mg GAE/g DW) compared to the cores (360.6 mg GAE/g DW) in the ethanolic extract. This finding supports our hypothesis that the petal fraction is superior to the core. However, the TPC values reported in their study are higher than those obtained in our research, likely due to the advanced ultrasound extraction technique employed, which tends to be more efficient than the conventional maceration method used in our study.

This encourages us to continue our research by evaluating the bioactivities to understand the factors contributing to the petal fraction’s superiority across all species.

### 2.4. Bioactivities

The significant difference in extraction yields and chemical composition between species, along with the superior yield of the petals fraction compared to the cores, led to further investigations into their biological properties. 

The goal was to assess their biological properties and determine how these differences influence their potential applications.

#### 2.4.1. Antioxidant Activity

**Figure 2:**
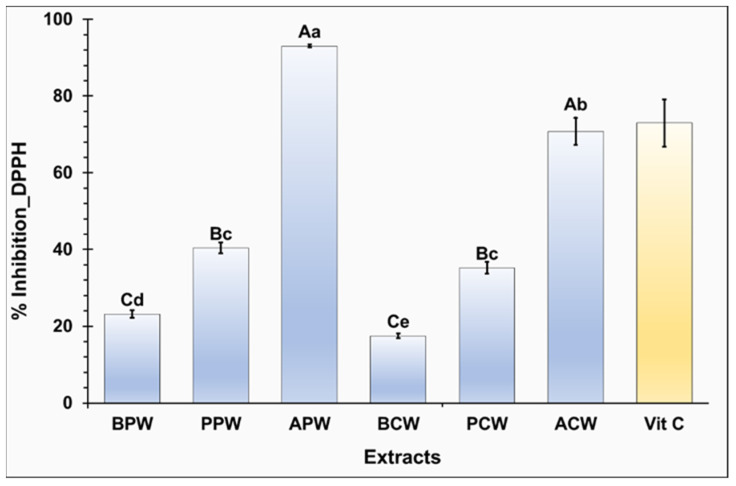
Antioxidant activity (%) against DPPH free radical. APW: *P. halepensis* Mill. petal water extract; ACW: *P. halepensis* Mill. core water extract; BPW: *P. brutia* Ten. petal water extract; BCW: *P. brutia* Ten. core water extract; PPW: *P. pinea* L. petal water extract; PCW: *P. pinea* L. core water extract. Each letter on the table represents a significant difference (*p* ≤ 0.05). Uppercase and lowercase letters mean a significant difference in species and fractions, respectively. Results are mean ± SD (n = 3).

Plant byproducts are a promising natural source of antioxidants. They produce antioxidative compounds to survive and neutralize reactive oxygen species (ROS) [20]. The antioxidant potential of six extracts (APW, ACW, BPW, BCW, PPW, and PCW) at 50 µg/mL was employed using the DPPH test and is illustrated in Figure 2. Vitamin C, as a reference, was used at 4 µg/mL and showed an inhibition of 73.24% against DPPH. 

Statistically, a significant difference in antioxidant activity was observed between extracts based on species and fractions (*p* ≤ 0.05). For all extracts, petal extracts showed higher antioxidant activity than core extracts (*p* ≤ 0.05) Notably, only the *P. halepensis* Mill. pine extracts demonstrated strong antioxidant potential, with inhibition rates of 93.05 and 70.78% for APW and ACW, respectively. However, *P. pinea* L. and *P. brutia* Ten. extracts displayed moderate antioxidant activity against DPPH, with inhibition values ranging from 20 to 40%.

The IC_50_ was determined from the curve, illustrating the relationship between inhibitory activity (%) and concentrations (µg/mL), to evaluate the efficacy of APW and ACW extracts in their antioxidant activity. A lower IC_50_ value indicates higher antioxidant potential and greater efficacity in neutralizing free radicals. 

As reported in Table 4, APW extract has better IC_50_ (13.51 µg/mL) than ACW extract (22.2 µg/mL) (*p* ≤ 0.05), confirming that petals are more interesting fractions than cores in antioxidant capacity.

Compared to the literature, the study of Belarbi [19] found that the IC_50_ in the aqueous extract of *P. halepensis* Mill. pinecones was 61.46 µg/mL, which is less interesting than the obtained values in the present study. This difference can be attributed to the importance of separating the petal and core, which can optimize the antioxidant potential of pinecones. Furthermore, the study of Costa et al. [16], using ultrasound, confirmed that the petal has a higher antioxidant potential (IC_50_ = 46.8 µg/mL) than the core (IC_50_ = 103.8 µg/mL) when a polar solvent is used, supporting our hypothesis that the petal fraction is superior to the core. However, these values are less impressive than those obtained in our study using aqueous maceration, possibly due to the intense mechanical forces and heat generated during the ultrasound process, which may have degraded or deactivated certain bioactive compounds.

#### 2.4.2. Anticancer Activity

This study marks the first exploration into anticancer activity using aqueous extracts from APW, ACW, BPW, BCW, PPW, and PCW. The anticancer activity of the six extracts at 50 µg/mL was tested against HepG2 and HeLa cancer cell lines, and the toxicity effect was assessed using the HEK-293 normal cell line (Table 5). 

All extracts were prepared at 50 µg/mL concentration and evaluated using MTT assay. Tamoxifen, a known cytotoxic agent, was used as a positive control.

As reported in Table 5, the results showed a notable reduction in the viability of HepG2, HeLa, and HEK-293 cells upon treatment with tamoxifen, confirming the reliability of the experimental method used (*p* ≤ 0.05). *P. halepensis* Mill. extracts showed the highest inhibition of cell growth, followed by *P. pinea* L. and *P. brutia* Ten. extracts against HeLa and HepG2 (*p* ≤ 0.05). However, petal and core extracts present no significant differences in inhibitory effect. APW and ACW extracts exhibited the highest inhibitory effect against HeLa cell lines (up to 62%) and HepG2 cell lines (57.74 and 47.22%, respectively). The differences in the inhibition of extracts against HeLa and HepG2 cells are attributed to the varying sensitivities of the cell lines, with HeLa cells being more sensitive than HepG2 cells. This variation in cell sensitivity has also been observed in previous studies [21,22].

HEK-293 cells are a non-cancerous cell line originally derived from human embryonic kidney cells. They were transformed by introducing fragments of sheared adenovirus type 5 DNA [23]. As an immortalized cell line, HEK-293 cells can divide indefinitely, making them very useful and widely used in biomedical research [24]. Evaluating these cells involves assessing their viability, proliferation, and any potential adverse effects caused by various compounds. This analysis is essential for understanding the safety profile of these compounds, particularly in healthy cell lines like HEK-293, which is key to determining their potential therapeutic applications. 

The results showed that all extracts exhibited low inhibition against HEK-293 cells, with values ranging from 8.88% to 14.94%. These values were significantly lower (at least four times less) than the inhibition values observed in the cancer cell lines HeLa and HepG2, confirming the lack of significant toxicity in our extracts.

In comparison with the literature, the result of the anticancer activity of pinecones from *Pinus yunnanensis* Franch in the study of Li et al. [25] demonstrated a significant potential with 73% inhibition against the HepG2 cell line. Additionally, the result of the anticancer activity of procyanidin bark extracts from *Pinus koraiensis Siebold and Zucc* in the study of Li et al. [26], reported an IC_50_ value of 196.38 µg/mL against HeLa cells. Furthermore, pinecones have been traditionally used in Japanese folk medicine (particularly for treating gastric cancer) [27,28], and have been employed to moisten the lungs, alleviate coughing, and reduce fever [29,30], confirming their non-toxic character.

#### 2.4.3. Antidiabetic Activity (α-Glucosidase)

α-glucosidase is a crucial enzyme in carbohydrate metabolism and is a clinical therapeutic target for regulating postprandial hyperglycemia [31]. Consequently, postprandial hyperglycemia can be managed by delaying glucose release through the inhibition of α-glucosidase activity [32]. 

In this context, aqueous extracts (APW, ACW, BPW, BCW, PPW, and PCW) have been tested to evaluate their ability to inhibit α-glucosidase (Table 6). 

Acarbose, a commercially available α-glucosidase inhibitor, was used as a standard for method validation. The development of acarbose as an α-glucosidase inhibitor represents an innovative approach to diabetes management. By competitively and reversibly inhibiting intestinal α-glucosidase, acarbose slows down carbohydrate digestion, prolongs the overall duration of this digestion, and thus reduces glucose absorption rates. Following oral administration of acarbose, postprandial blood glucose increase is reduced dose-dependently, and glucose-induced insulin secretion is attenuated. Due to the reduction in postprandial hyperglycemia and hyperinsulinemia induced by acarbose, triglyceride absorption in adipose tissue, hepatic lipogenesis, and triglyceride content are also reduced. Consequently, acarbose treatment not only smooths out the postprandial blood glucose curve due to its primary and secondary pharmacodynamic effects but also improves overall metabolic status [33].

As shown in Table 5, at 50 µg/mL, only the APW extract showed significant α-glucosidase inhibition (77.20%). Acarbose at the same concentration, used as a reference to confirm the method, showed an inhibition of 78.42%. However, *P. brutia* Ten. and *P. pinea* L. extracts did not show any inhibition potential for antidiabetic activity against α-glucosidase, highlighting the importance of species selection.

The IC_50_ of APW was determined from the curve showing the relationship between inhibitory activity and concentrations. A lower IC_50_ value indicates a higher inhibitory potential of the extract against α-glucosidase. 

As detailed in Table 6, the APW extract demonstrated an interesting IC_50_ value of 24.70 µg/mL, highlighting its high potential for antidiabetic activity against α-glucosidase.

No previous studies have investigated the antidiabetic activity of petal and core extracts from *Pinus* species. Liu et al. [34] demonstrated that pine bark extract, rich in Pycnogenol, strongly inhibits α-glucosidase, highlighting its effectiveness in treating type 2 diabetes. Similarly, Dakhlaoui et al. [35] found that essential oils from *P. halepensis* Mill. pine needles inhibit α-glucosidase with an IC_50_ of 254.45 µg/mL. However, our results are more promising, likely due to the specific use of petal and core fractions, as well as differences in extraction techniques.

The promising bioactivities of the extracts motivate us to continue our research by identifying the bioactive compounds in each extract to better understand the factors contributing to the differences between species and the petal fraction’s superiority across all species.

### 2.5. Identification of Bioactive Compounds

The significant differences in extraction yields and chemical composition, and the potential bioactivities between species, along with the superior yield of the petals fraction compared to the cores, have led to further investigations into the identification of bioactive compounds from extracts studied.

#### 2.5.1. Identification of Organic Non-Volatile Compounds by HPLC

The non-volatile compounds found in APW, ACW, BPW, BCW, PPW, and PCW were identified using the HPLC-DAD technique and reported in Table 7. 

Phytochemical analysis of extracts using HPLC-DAD was conducted to identify 14 compounds, of which 1 (Trihydroxyethylrutin) was detected for the first time in *Pinus*. The *P. pinea* L. extracts revealed the presence of eight compounds, whereas *P. halepensis* Mill. and *P. brutia* Ten. extracts contained seven and six molecules, respectively. 

For all species, petal extracts are richer in chemical compounds than core extracts, which could justify the superiority of this fraction on yields and bioactivities. APW extract contains the highest content of trihydroxyethylrutin (143.65), known for its biological properties [36], which could estimated to be responsible for the superiority of APW extract on antioxidant, anticancer, and antidiabetic potentials compared to others. The chemicals sinapic acid and trans-3-hydroxycinnamic acid, known for their biological properties [37,38], were exclusively detected in APW (2.33 and 3.45 respectively), which could make this extract especially valuable for antioxidant, anticancer, and antidiabetic activities compared to others extracts. 5-hydroxy-4′-methoxylflavone, gallic acid, and gallocyanin were identified only in PPW and PCW extracts, and are known for their antioxidant, anti-inflammatory, and anticancer potential [39], which could explain the observed biological properties in these extracts. 4-hydroxy-3-(3-oxo-1-phenylbutyl)coumarin was identified only in both *P. pinea* L. (0.38 and 0.47 for PCW and PPW, respectively) and *P. halepensis* Mill. (4.66 for ACW) extracts, and known for anticoagulant, antibacterial, anti-inflammatory, antioxidant, antitumor, antiviral, and enzyme inhibition properties [39,40], which could explain the highly biological properties of these species. Various compounds, including catechin, chlorogenic acid, and trihydroxyethylrutin, known for their biological properties, were detected in all species studied, suggesting common characteristics between these species [36].

Compared to the literature, Vinothkumar et al. [36] demonstrated the chemopreventive potential of trihydroxyethylrutin in DMH-induced colon carcinogenesis in rats, particularly at a dose of 25 mg/kg, which showed significant beneficial effects. These findings support the anticancer activity observed in our extracts, indicating that trihydroxyethylrutin may be useful in cancer prevention. The catechin was previously identified in *P. halepensis* Mill. pinecones by Kilic Pekgözlü et al. [41] and Meziti et al. [42]. Furthermore, chlorogenic acid was identified in *P. koraiensis* cones by Wang et al. [43].

**Table 7:** Identification of non-volatile compounds by HPLC-DAD.

N°	Compounds	RT (min)	Structure	Peak Area mAU·min						References
				ACW	APW	BCW	BPW	PCW	PPW	
**1**	Trihydroxyethylrutin	0.86	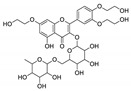	4.87	143.65	2.94	82.43	5.03	103.62	[36]
**2**	Catechin	0.90	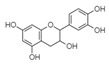	20.36	4.70	0.77	-	6.65	-	[44]
**3**	Ellagic acid	0.96	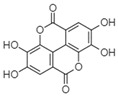	-	-	-	199.79	1.46	208.08	[45]
**4**	Chlorogenic acid	1.19	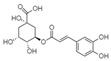	-	56.77	3.18	-	3.65	-	[46]
**5**	Gallic acid	2.05	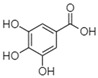	-	-	-	-	2.01	-	[47]
**6**	Gallocyanin	2.34	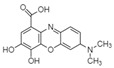	-	-	-	-	0.27	0.17	[48]
**7**	Methyl 3,5-dihydroxybenzoate	2.52	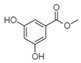	-	-	0.49	-	-	-	[48]
**8**	Sinapic acid	3.33	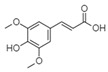	-	2.33	-	-	-	-	[49]
**9**	Trans-3-hydroxycinnamic acid	3.65	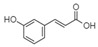	-	2.45	-	-	-	-	[48]
**10**	Trans-ferulic acid	4.29	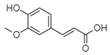	1.77	-	-	-	-	-	[50]
**11**	5-hydroxy-4′-methoxylflavone	18.70	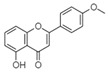	-	-	-	-	0.38	0.31	[48]
**12**	Chrysin	18.92	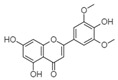	-	-	-	11.99	-	-	[51]
**13**	4-hydroxy-3-(3-oxo-1-phenylbutyl)coumarin	19.10	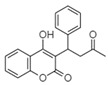	4.66	-	-	-	0.38	0.47	[48]
**14**	3′-hydroxy-a-naphthoflavone	19.37	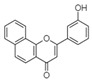	0.08	-	-	11.99	-	-	[48]

APW: *P. halepensis* Mill. petal water extract; ACW: *P. halepensis* Mill. core water extract; BPW: *P. brutia* Ten. petal water extract; BCW: *P. brutia* Ten. core water extract; PPW: *P. pinea* L. petal water extract; PCW: *P. pinea* L. core water extract; “-“: not detected; RT: retention time.

#### 2.5.2. Identification of Organic Volatile Compounds by GC-MS

The identified compounds in the different extracts, using GC-MS analysis before and after derivatization, are reported in Table 8 and Table 9, respectively. 

GC-MS analysis was initially performed to detect nine compounds in their natural state. Derivatization was then used to enhance the identification of additional compounds (for example, dense compounds), where the number of identified compounds expanded to 28, indicating the significant sensitivity and accuracy of the derivatization method. Eleven of the identified compounds were found for the first time in these species, including 2-phenyltetralin, 1,2-diphenylcyclobutane, 1-phenyltetralin, 1,3,5-triphenylcyclohexane, p-toluic acid, 1-dodecanol, xylitol, d-pinitol, α-d-fructofuranose (isomer1), α-d-(+)-talopyranose isomer 2, and β-d-xylopyranose.

*P. halepensis* Mill. and *P. pinea* L. extracts revealed the presence of 19 compounds, whereas the *P. brutia* Ten. extracts contained 18 molecules. Petal extracts are richer in chemical compounds for all species than core extracts, which could explain the superiority of petal extracts in biological properties. Notably, 1-phenyltetralin and 1,3,5-triphenylcyclohexane were found only in APW and ACW extracts with a surface area of up to 1.44 × 10^7^, which could be attributed to the specified biological properties of these extracts like antioxidant, anticancer, and antidiabetic potentials. However, the remaining compounds were shared between the extracts of the studied species, such as 2,4-di-tert-butylphenol, 2,2′-methylenebis[6-(1,1-dimethylethyl)-4-methyl-, 2-phenyltetralin, 1,2-diphenylcyclobutane, lactic acid, glycerol, α-d-fructofuranose (isomer1), palmitic acid, and stearic acid. The common presence of these compounds in all extracts could provide valuable insights into the chemical composition of these species and their potential applications in various fields.

All extracts demonstrated richness in organic acids and alcohol compounds as indicated by their substantial surface area, well-known for antimicrobial, antioxidant, anticancer, and anti-inflammatory activities, making the studied extracts a promising candidate for future drug development [52]. APW extract, with its high anticancer, antioxidant, and antidiabetic potential, as detailed in the previous sections (Section 2.4.1, Section 2.4.2 and Section 2.4.3), is rich in d-pinitol (439 × 10^7^), α-D-(+)-talopyranose isomer2 (115 × 10^7^), and palmitic acid (75 × 10^7^), known for its diverse pharmacological activities and significant therapeutic potential for treating cancer, diabetes, and oxidative stress [53]. Therefore, these molecules are estimated to be responsible for the important antioxidant, anticancer, and antidiabetic activities observed in APW extract. Based on data from PubChem and DrugBank, glycerol, found in our APW, ACW, BCW, BPW, and PCW extracts (with high surface areas of 173, 59, 578, 219, and 153 × 10^7^, respectively), is non-toxic to human skin and has a low toxic dose of 1428 mg/kg when taken orally [54], as well as being widely used as a sweetener and moisturizer in food, a skin protectant in cosmetics, and an ingredient in pharmaceuticals [55]. The presence of glycerol in our extracts likely explains their biological effects and supports their safe use in food, pharmaceuticals, and cosmetics.

In comparison to previous studies, palmitic acid and stearic acid were found in the seeds and cones of *P. halepensis* Mill. pine [3]. Additionally, 49 volatile compounds, including caryophyllene oxide and bornyl acetate, were identified in various parts of *P. halepensis* Mill. pine, such as needles, twigs, and buds [56].

**Table 8:** Identification of volatile compounds before derivatization by GC-MS.

				Area (×10^7^)						
N°	Compounds	RTmin	Structure	ACW	APW	BCW	BPW	PCW	PPW	References
**1**	Bicyclo [2.2.1]heptane-2,5-diol, 1,7,7-trimethyl-, (2-endo,5-exo)-	14.19	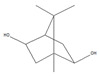	-	-	-	-	13.3	-	[48]
**2**	Limonene glycol	14.49	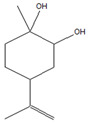	-	-	-	12.27	63.3	14.6	[48]
**3**	Cetyl alcohol	15.5	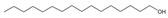	-	2.41	1.30	-	-	-	[48]
**4**	2,4-di-tert-butylphenol	16.33	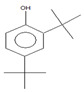	5.34	5.52	3.95	6.16	4.26	4.88	[48]
**5**	Phenol, 2,2′-methylenebis[6-(1,1-dimethylethyl)-4-methyl-	17.92	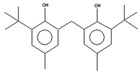	3.73	3.51	2.87	-	-	-	[48]
**6**	2-phenyltetralin	19.21	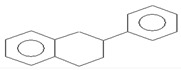	8.23	10.2	-	13.7	-	11.1	
**7**	1,2-diphenylcyclobutane	19.67	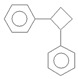	335	65.2	-	76.6	27	76.7	
**8**	1-phenyltetralin	20.03	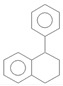	0.97	1.44	-	-	-	-	
**9**	1,3,5-triphenylcyclohexane	20.41	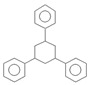	-	0.77	-	-	-	-	

APW: *P. halepensis* Mill. petal water extract; ACW: *P. halepensis* Mill. core water extract; BPW: *P. brutia* Ten. petal water extract; BCW: *P. brutia* Ten. core water extract; PPW: *P. pinea* L. petal water extract; PCW: *P. pinea* L. core water extract; “-“: not detected; RT: retention time.

**Table 9:** Identification of volatile compounds after derivatization by GC-MS.

N°	Compounds	RTmin	Structure	ACW	APW	BCW	BPW	PCW	PPW	References
**1**	Glycol	7.13	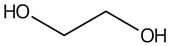	4	6.39	-	-	30.8	-	[48]
**2**	glycol propyl	7.29	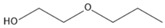	4.45	-	-	-	-	-	[48]
**3**	Lactic acid	8.74	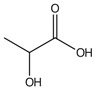	-	7.64	26.5	34.3	16.4	-	[57]
**4**	3-hydroxybutyric acid	10.06	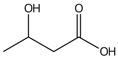	-	-	3.76	-	-	-	[48]
**5**	Glycerol	11.7	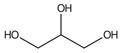	59.1	173	578	219	153	-	[48]
**6**	Succinic acid	13.06	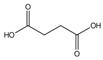	-	-	11.54	-	10.5	-	[48]
**7**	Catechol	13.25	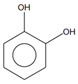	-	-	-	-	19.9	-	[48]
**8**	Citric acid	14.01	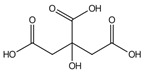	-	-	-	42.3	-	-	[57]
**9**	p-toluic acid	14.27	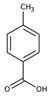	10.7	-	-	-	18.2	-	
**10**	1-dodecanol	16.01	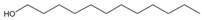	21	-	-	-	-	-	
**11**	D-(-)-ribofuranose	17.22	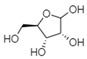	-	-	-	147	19.9	-	[48]
**12**	Xylitol	17.4	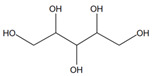	-	-	-	342	-	-	
**13**	d-pinitol	18.65	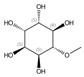	-	439	19,5	112	557	-	
**14**	α-d-fructofuranose (isomer1)	18.73	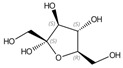	-	9.86	10.2	-	8.41	-	
**15**	Protocatechuic acid	19.1	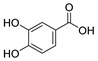	-	33.7	-	-	87.8	-	[58]
**16**	α-D-(+)-talopyranose isomer2	19.35	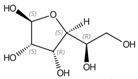	-	115	-	-	182	-	
**17**	β-d-xylopyranose	19.64	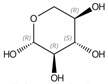	-	-	148	-	147	-	
**18**	Palmitic acid	20.94	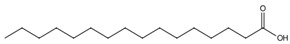	-	75.2	60.3	116	67.5	-	[59]
**19**	Stearic acid	23.35	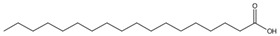	-	22.7	-	21.1	13.1	-	[59]

APW: *P. halepensis* Mill. petal water extract; ACW: *P. halepensis* Mill. core water extract; BPW: *P. brutia* Ten. petal water extract; BCW: *P. brutia* Ten. core water extract; PPW: *P. pinea* L. petal water extract; PCW: *P. pinea* L. core water extract; “-“: not detected; RT: retention time.

### 2.6. Principal Component Analysis (PCA)

**Figure 3:**
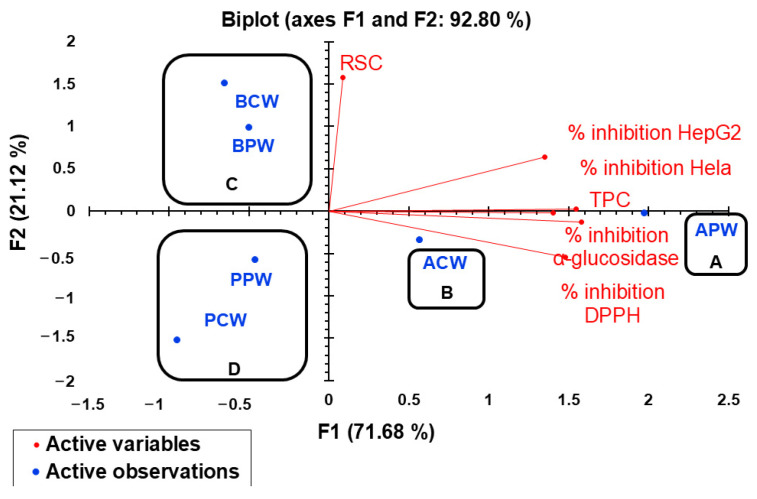
Principal component analysis.

Principal component analysis (PCA) was used to elucidate the connections among six key components, namely, TPC (total polyphenolic compounds), RSC (reducing sugar content), % inhibition DPPH (antioxidant activity against DPPH), % inhibition HeLa (anticancer activity against HeLa cell line), % inhibition HepG2 (anticancer activity against HepG2 cell line), and % inhibition α-glucosidase (antidiabetic activity) for APW, ACW, BPW, BCW, PPW, and PCW, and reported in Table 10 and Figure 3.

As illustrated in Figure 3, the first two principal components (F1 and F2) encompassed a substantial 92.80% of the data variability. The primary axis (F1) was strongly positively correlated with TPC, % inhibition DPPH, % inhibition HepG2, % inhibition HeLa, and % inhibition α-glucosidase with correlation coefficients R^2^ of 0.99, 0.93, 0.85, 0.97, and 0.88, respectively. F2 was only correlated with RSC (R^2^ = 0.99). As shown in Figure 3, the six extracts exhibit clear segregation into four principal groups (A, B, C, and D). Group A comprises only APW extract, group B includes only ACW extract, group C encompasses all *P. brutia* Ten. extracts, and group D includes all *P. pinea* L. extracts. Notably, extracts in group A were characterized by the highest chemical analysis and bioactivities. Group B is characterized by high chemical analysis and bioactivities but with less significance than Group A. Group C encompasses *P. brutia* Ten. extracts, demonstrating a richness in reducing sugars, and Group D encompasses the remaining extracts. This grouping highlights the varying bioactivity profiles of the extracts from different pine species and fractions.

Considering ACP analysis, the aqueous *P. halepensis* Mill. petal extract (APW extract) could be identified as the most interesting extract, due to its richness in TPC and its antioxidant, anticancer, and antidiabetic activities.

Compared to the literature, the correlation between polyphenol content and antioxidant activity have been explored in different previous studies on plant extracts. Ait Atmane et al. [45] found a strong correlation between TPC and DPPH in *P. halepensis* Mill. seeds with R^2^ = 0.95.

## 3. Materials and Methods

### 3.1. Chemical

All chemicals used were of analytical reagent grade. All reagents and analytical standards were purchased from Sigma Aldrich (Saint-Quentin, France): acarbose, methanol, DMSO, DPPH, DNSA, Folin–Ciocalteu reagent (2N), gallic acid, HCl, KH_2_PO_4_, MTT, NaOH, Na_2_HPO_4_, sodium carbonate, tamoxifen, PNP-G, and phosphate buffer. The analytical standards used to identify the non-volatile compounds present in the extracts were trihydroxyethylrutin; rutin; catechin; 3,4-dihydroxy-5-methoxybenzoic acid; quercetin, 3-β-D-glucoside; polydatin; 2,4-dihydroxycinnamic acid; ellagic acid; (±) synephrine; chlorogenic acid; gallic acid; (−)-epicatechin; gallocyanine; brilliant yellow; methyl 3,5-dihydroxybenzoate; 3-amino-4-hydroxybenzoic acid; 3,4-dihydroxycinnamic acid (caffeic acid); sinapic acid; trans-3-hydroxycinnamic acid; p-coumaric acid; trans-ferulic acid; 4,7-dihydroxycoumarin; 7-hydroxycoumarin-3-carboxylic acid; 7-hydroxycoumarin-3-carboxylic acid; methyl 4-hydroxybenzoate; myricetin; 6-hydroxycoumarin; coumarin; 7-hydroxy-4-methyl-3-coumarinylacetic acid; 3-cyanoumbelliferone; isopropyl 3,4,5-trihydroxybenzoate; resveratrol; 4-hydroxy-3-methoxycinnamic acid; 2-hydroxycinnamic acid; quercetin; ethyl 3,4-dihydroxycinnamate; 7,8-dihydroxy-2,2-dimethylchromane-6-carboxylic acid; trans-cinnamic acid; 3,4-dihydroxybenzoic acid methyl ester; 4-ethyl-7-hydroxy-8-methyl-2H-chromen-2-one; α-cyano-4-hydroxycinnamic acid; naringenin; (±)-taxifolin; 7,3′-dihydroxyflavone; 2,4-dihydroxy-3,6-dimethylbenzoic acid; 3-cyano-7-hydroxy-4-methylcoumarin; (±)-6-hydroxy-2,5,7,8-tetramethylchroumane-2-carboxylic acid; butyl gallate; 6-hydroxyflavone; baicalein; ethyl 3,5-dihydroxybenzoate; ethyl trans-2-hydroxycinnamate; kaempferol; 5,8-dihydroxy-1,4-naphthoquinone; ethyl 4-hydroxy-3-cinamate; 7-hydroxy-4-phenylcoumarin; 2-chloro-3-(4-hydroxy-phenylamino)-(1,4) naphthoquinone; 5-hydroxy-4′-methoxylflavone; chrysin; warfarin (4-hydroxy-3-(3-oxo-1-phenylbutyl)coumarin); icariin; 3′-hydroxy-a-naphthoflavone; 3-tert-butyl-4-hydroxybenzoic acid; 5,7-dihydroxy-3′,4′,5′-trimethoxyflavone; 7-hydroxyflavone; β-carotene; lutein; 4-hydroxytamoxifen; 5,7-dihydroxy-4-propyl-coumarin; shikonin; 3′-hydroxy-6-methylflavone; 7-hydroxy-4-(trifluoromethyl) coumarin; 5-hydroxyflavone; isobutyl 4-hydroxybenzoate; 3,3′,4′-trimethoxyflavone; butyl 4-hydroxybenzoate; 7-hydroxy-3′,4′,5′-trimethoxy-alpha-naphthoflavone; 3′-hydroxy-b-naphtoflavone; 3,3′-dimethoxyflavone; 2,3-dichloro-5,8-dihydroxy-1,4-naphthoquinone; 3,6,3′-trimethoxyflavone; 3,7-dimethoxyflavone; 5-hydroxy-3′-metho-xyflavone; xanthurenic acid; 4′,5′-dimethoxy-2′-hydroxy-4-methylchalcone; rosmarinic acid; (z)-3-(3-ethoxy-4-hydroxy-phenyl)-2-phenyl-acrylic acid; 2-chloro-3-(3,5-di-tert-bu-tyl-4-hydroxy-phenyl)(1-4)naphtoquinone; hamamelitannin; 3,4-dihydroxy-5-methoxycinnamic acid; and 5-hydroxy-7-((3-methylbenzyl)oxy)-2-phenyl-4h-chromen-4-one.

### 3.2. Sample Preparation

The collection, identification, and preparation of plant materials were conducted following the methodology described in our previous publication [48]. Briefly, mature pinecones from *P. halepensis* Mill., *P. brutia* Ten., and *P. pinea* L. pines were initially collected in Bizerte (northern Tunisia) in December 2016. The pinecones utilized had undergone sun drying in their natural environment before collection, as they were gathered from the ground. They were then divided into two fractions, petal and core, which were separated manually and milled [48].

Previous studies have generally analyzed the whole pinecone without separating its components. In contrast, we divided the cone into petals (scales) and core to explore potential differences in their biochemical properties, employing an environmentally friendly and green process for both separation and extraction of bioactive compounds. This approach enables a more detailed and sustainable analysis.

### 3.3. Moisture Content

To determine the moisture content of the materials, empty crucibles were first dried in an oven (Thermo Fisher Scientific, ref: 0562202010) at 105 °C for 3 h and weighed. A 1 g sample of each material was then dried in the oven for 24 h at 105 °C, and the combined weight of the crucible and dried sample was recorded, as described by Barssoum et al. [60]. Based on these measurements, absolute humidity (x) in % (grams of water per gram of dry material) (% g water/g dm) and relative humidity (n) in % grams of water per gram of humid material (% g water/g hm) were calculated, as shown in Equation (1):

(1) $$humidity\left(n\;or\;x\right)=\frac{m_{water}}{m_{material\;}\left(dry\;or\;humid\right)}$$

### 3.4. Particle Size

The mean diameter of each part was performed ex-situ using a morphogranulometer (Mastersizer G3S, Ltd. SN: MAL1033756, software Morphologi v7.21), as described by Barssoum et al. [60]. This optical device includes a system of lens (magnification: from ×1 to ×50, dimension min/max: 0.5/3000 μm) and a camera (Nikon CFI60) with a resolution close to 0.06 μm/pixel. The analyses were conducted in a dry way by dispersing substrate (mass: 5 mg) through a dispersion system unit (DSU) (pressure: 4 bar, 30 ms, settling 60 s). Image acquisition and analysis were performed according to a standard operating procedure (SOP): diascopic light, bright mode, light intensity (80% ± 0.2), magnification (×10), threshold (Th = 136), and scanned area (10 × 10 mm). The morphogranulometer system scanned and recorded the images. Image analysis software (Morphologi v7.21) allowed for identifying individual particles and calculating their geometrical properties (diameter, aspect ratio, circularity, length, and width).

### 3.5. Extraction

Each sample (5 g) was extracted by maceration in 50 mL distilled water for 24 h at 20 °C with constant stirring at 150 rpm. Distilled water was selected as a non-toxic and green solvent. The low-temperature, prolonged extraction optimized the solubilization of bioactive compounds while minimizing energy consumption and protecting sensitive compounds from thermal degradation, ensuring an eco-friendly extraction process. The mixtures were then filtered and concentrated by distillation in a rotary evaporator (IKA, RV 10 auto V, Staufen, Germany) under vacuum at reduced pressure and a temperature of 35 °C.

The extraction yields obtained for the six aqueous extracts from three *Pinus* petal fractions, *P. halepensis* Mill., *P. brutia* Ten., and *P. pinea* L. (APW, BPW, and PPW, respectively), and the core fractions of the same species (ACW, BCW, and PCW respectively) were determined in % (*w/w*).

### 3.6. Analyses of Reducing Sugar Content

Quantification of reducing sugars (RSC) in aqueous extracts (APW, BPW, PPW, ACW, BCW, and PCW) was performed according to the 3,5-dinitrosalicylic acid (DNSA) method, as described in our previous publication [48]. First, 150 μL of each extract (350 mg/L) was added to 150 μL of DNS solution. Then, 750 μL of deionized water was added after incubation at 100 °C for 5 min with constant stirring. The absorbance of the mixture was measured at 530 nm. This measurement was performed using a reference blank (sodium potassium tartrate in NaOH 2 M instead of DNSA) and negative control (where the extracts were replaced by dimethyl sulfoxide (DMSO).

The sugar content was determined in milligrams of glucose equivalent per gram of dry extract (mg GE/g DW).

### 3.7. Analyzes of Total Phenolic Content

The total phenolic content (TPC) in aqueous extracts (APW, BPW, PPW, ACW, BCW, and PCW) was estimated with a colorimetric assay using the Folin–Ciocalteu method, as described in our previous publication [48]. In an alkaline environment created by the sodium carbonate (Na_2_CO_3_), the phenolic compounds in the samples were oxidized, leading to the reduction of the Folin–Ciocalteu reagent’s phosphotungstic and phosphomolybdic acids into a blue-colored complex. The intensity of this blue color, which corresponds to the content of phenolic compounds present, was measured using a microplate reader (Multiskan Go, F1-01620, Thermo Fisher Scientific, Vantaa, Finland) at 765 nm.

TPC content was determined as milligrams of gallic acid equivalents per gram of dry weight (mg GAE/g DW) based on the regression equation obtained from the standard calibration curve of gallic acid concentrations ranging from 0 to 115 mg/L.

### 3.8. Determination of Antioxidant Activity

The antioxidant activity of the six aqueous extracts (APW, BPW, PPW, ACW, BCW, and PCW) was determined against 1,1-diphenyl-2-picrylhydrazyl (DPPH), as described in our previous publication [48]. The ascorbic acid was used as a reference at 4 µg/mL. A quantity of 20 µL of each extract at a concentration of 0.5 mg/mL was combined with 180 µL of a methanolic DPPH solution (0.2 N) and incubated at 25 °C for 25 min in a 96-well microplate (Micro Well; Thermo Fisher Scientific, Illkirch, France). Absorbance was then measured at 524 nm using a microplate reader (Multiskan Go F1-01620, Thermo Fisher Scientific, Vantaa, Finland).

The percentage inhibition of DPPH was determined by Equation (2):

(2) $$\%inhibition=100\times \frac{A_{blanc}-A_{sample}}{A_{blanc}}$$

where $A_{blanc}$ is the absorbance of the solvent and DPPH radical when no samples are present and $A_{sample}$ is the absorbance of the sample and DPPH radical.

### 3.9. Determination of Anticancer Activity

The anticancer activity of the aqueous extracts (APW, BPW, PPW, ACW, BCW, and PCW) was employed using the 3-(4,5-dimethyl-thiazol-2-yl)-2,5-diphenyltetrazolium bromide (MTT) test as described in our previous publication [48]. Both cancer cell lines were tested: a human liver cancer cell line (HepG2) and a human epithelial cervix carcinoma (HeLa). The toxicity effect of extracts was assessed in human embryonic kidney cells (HEK-293). Tamoxifen, as a reference, was used at 1, 10, and 100 μM. HepG2 (ATCC HB-8065) and HeLa cell lines (ATCC CRM-CCL-2) were grown in Advanced DMEM (Thermo Fisher Scientific), while HEK-293 cells (ATCC CRL-1573) were maintained in high-glucose DMEM (Dulbecco’s Modified Eagle’s Medium, France). Each culture medium was supplemented with 10% decomplemented fetal bovine serum, 1% non-essential amino acids, and antibiotics, including penicillin, streptomycin, and gentamicin. Cell cultures were kept in a humidified incubator at 37 °C with 5% CO_2_. When cells reached 70–80% confluence, they were collected for cytotoxicity assays. Adherent cells were seeded into 96-well plates at a density of 12,000 cells/well for HepG2, HeLa, and HEK-293 lines, and the plates were incubated overnight at 37 °C in a humidified atmosphere with 5% CO_2_. Cells were then treated in triplicate with each extract diluted to 50 µg/mL and incubated for 48 h at 37 °C. After removing the supernatant, 50 μL of MTT solution was added to each well, and plates were incubated at 37 °C for 40 min. Following incubation, the MTT solution was discarded, and the dark-blue formazan crystals, formed by the reduction of yellow MTT by mitochondrial dehydrogenases in viable cells, were dissolved in 80 μL of DMSO. Absorbance at 605 nm was then recorded using a microplate reader (Mullikan Go, F1-01620, Thermo Fisher Scientific, Vantaa, Finland).

### 3.10. Determination of Antidiabetic Activity

The antidiabetic activity, through the inhibition of α-glucosidase by various aqueous extracts (APW, BPW, PPW, ACW, BCW, and PCW) was evaluated using the PNP-G (p-nitrophenyl glucopyranoside) method as detailed by Ben Khadher et al. [13]. In the reaction mixture, 50 μL of phosphate buffer (0.1 M, pH 6.9) was combined with 100 μL of α-glucosidase enzyme (1 U/mL) and 50 μL of each extract (0.5 mg/mL). After 10 min incubation at 25 °C, 50 μL of PNP-G (5 mM) was added. Following another 5 min incubation, the absorbance was measured at 405 nm. Enzyme inhibition was calculated as described in Equation (2).

### 3.11. Identification of Non-Volatile Compounds (HPLC)

The non-volatile compounds of the aqueous extracts (APW, BPW, PPW, ACW, BCW, and PCW) were identified using HPLC-DAD (Thermo Scientific Accela pump, Accela PDA detector), as described in our previous publication [48]. The separation was performed on an RP-C18 column (Phenomenex; Le Pecq, France, 25 cm × 4.6 mm, 5 μm) with a flow rate set at 0.5 mL/min. The mobile phase included acidified water (pH = 2.65) as solvent A, and a mixture of acidified water and acetonitrile (H_2_O/ACN, 20/80 *v*/*v*) as solvent B. A linear gradient elution method was applied, increasing the concentration of solvent B from 12 to 30% over 15 min, then rising more quickly from 30 to 50% within 2 min, and ultimately reaching 99.9% in 3 min. The concentration was then decreased back to 12% B over 7 min.

The extracts were dissolved at 10 mg/mL in acidified water/ACN (20:80 *v*/*v*). Compound identification was employed by comparing their retention times and maximum absorbance (lambda max) values with those of reference standards at 280 nm, which were analyzed under the same conditions as the samples.

### 3.12. Identification of Volatile Compounds (GC-MS)

The volatile compounds of the aqueous extracts (APW, BPW, PPW, ACW, BCW, and PCW) were identified using GC-MS, as described in our previous publication [48]. Extracts were dissolved at 3 mg/mL in ACN. The analysis was performed on a Saturn 2000 gas chromatograph (Les Ulis, France) with a DB-5MS fused silica capillary column (5% phenylmethylpolysiloxane, 30 × 0.25 mm, 0.25 μm film thickness). Hydrogen served as the carrier gas. The column temperature program started at 60 °C for 1 min, then rose at 10 °C/min to 150 °C, where it was held for 1 min. A second gradient increased the temperature to 260 °C at 12 °C/min, held for 10 min. Mass spectrometry data were acquired in full-scan mode (70–800 AMU), with the ion source at 220 °C and the transfer line at 240 °C. 5 μL per extract were injected to analyze.

Compounds in the extracts were identified by comparing their mass spectra to those in the NIST08 database (National Institute of Standards and Technology, https://www.nist.gov/, MS library version 2.4, build 25 March 2020).

Derivatization method: 340 μL of the samples prepared as described above were added to 60 μL of N, O bis(trimethylsilyl)trifluoroacetamide (BSTFA) reagent and then incubated at 40 °C for 30 min. Spectral analysis of each derivative solution followed the procedure described in the previous section. Derivatization is an essential technique in analytical chemistry that enhances sensitivity and precision, particularly in GC and GC-MS. This technique involves chemically modifying analytes to improve their separation and detection capabilities. Derivatization selectively changes analytes without substantially affecting the overall sample matrix. For example, it replaces active hydrogens (for example polar and non-volatile) in functional groups such as OH, COOH, SH, NH, and CONH. These modifications increase the volatility of the compounds, which is crucial for their analysis by GC or GC-MS, where volatility is key for accurate measurement and identification [61].

### 3.13. Statistical Analysis

The experimental data were expressed as mean values with standard deviations, and each sample was measured in triplicate. The difference between the species and the fractions was evaluated using the Tukey test. Principal Component Analysis (PCA) was employed using XLSTAT (version 2021.3.1, Addinsoft, Pearson edition, Waltham, MA, USA).

## 4. Conclusions

This study demonstrates that aqueous extracts from *Pinus* species, specifically *P. halepensis* Mill., *P. brutia* Ten., and *P. pinea* L., hold significant potential not only for medical and industrial applications but also for circular economy and sustainability. Using water as a green and natural solvent in maceration supports a sustainable and environmentally friendly extraction process.

All extracts are rich in chemical composition and exhibit interesting bioactivities. Notably, they effectively inhibited cancer cell lines (HeLa and HepG2) while showing low toxicity to normal HEK-293 cells, indicating their safety for therapeutic use.

Among these, the extract from *P. halepensis* Mill. petals (APW) stands out due to its high polyphenol content and pronounced bioactive properties, particularly its antioxidant activity and strong inhibition of α-glucosidase, suggesting its potential for diabetes treatment. The superior bioactivities of the petal extracts compared to the core extracts may be attributed to the specific phytochemicals present in the petals.

This research not only enhances our understanding of the phytochemical profiles of these *Pinus* species but also underscores the importance of utilizing both petals and cores in future studies.

Overall, these findings encourage further investigation into the health benefits and potential applications of *Pinus* species in the food, cosmetic, and pharmaceutical industries.

## Figures and Tables

**Table 1:** Moisture content and particle size of materials.

Samples	n % (g water/g hm)	x % (g water/g dm)	Mean Diameter (µm)
*P. halepensis* Mill. petal	9.70 ± 0.27	8.84 ± 0.23	94.77 ± 8.58
*P. halepensis* Mill. core	9.95 ± 0.13	9.05 ± 0.11	88.12 ± 12.2
*P. brutia* Ten. petal	11.06 ± 0.29	9.96 ± 0.24	89.98 ± 12.27
*P. brutia* Ten. core	10.05 ± 0.43	9.13 ± 0.36	83.22 ± 2.95
*P. pinea* L. petal	9.39 ± 0.17	8.59 ± 0.14	79.72 ± 5.58
*P. pinea* L. core	9.00 ± 0.08	8.26 ± 0.07	97.02 ± 7.51

n: relative humidity; x: absolute humidity; hm: humid materials; dm: dry materials.

**Table 2:** Extraction yields (%).

Extracts	Extraction Yields (%)
APW	2.47 ± 0.04 ^Bc^
ACW	2.02 ± 0.13 ^Bd^
BPW	3.93 ± 0.07 ^Aa^
BCW	1.77 ± 0.01 ^Ad^
PPW	1.58 ± 0.02 ^Ce^
PCW	1.01 ± 0.02 ^Cf^

APW: *P. halepensis* Mill. petal water extract; ACW: *P. halepensis* Mill. core water extract; BPW: *P. brutia* Ten. petal water extract; BCW: *P. brutia* Ten. core water extract; PPW: *P. pinea* L. petal water extract; PCW: *P. pinea* L. core water extract. Each letter on the table indicates a significant difference (*p* ≤ 0.05). Uppercase and lowercase letters mean a significant difference in species and fractions, respectively.

**Table 3:** Reducing sugar content (RSC).

Extracts	RSC (mg GE/g DW)
APW	325.12 ± 12.81 ^Bc^
ACW	261.50 ± 13.84 ^Bd^
BPW	404.55 ± 12.31 ^Ab^
BCW	470.09 ± 6.62 ^Aa^
PPW	222.83 ± 4.44 ^Ce^
PCW	158.31 ± 14.28 ^Cf^

APW: *P. halepensis* Mill. petal water extract; ACW: *P. halepensis* Mill. core water extract; BPW: *P. brutia* Ten. petal water extract; BCW: *P. brutia* Ten. core water extract; PPW: *P. pinea* L. petal water extract; PCW: *P. pinea* L. core water extract; GE: glucose equivalent; DW: dry weight. Each letter on the table indicates a significant difference (*p* ≤ 0.05). Uppercase and lowercase letters represent a significant difference in species and fractions, respectively. Results presented as mean ± SD (n = 3).

**Table 4:** IC_50_ of antioxidant activity against DPPH free radical.

Extracts	APW	ACW	Vit C
DPPH IC_50_ (µg/mL)	13.51 ± 0.23 ^a^	22.2 ± 1.91 ^b^	3.65 ± 0.03

APW: *P. halepensis* Mill. petal water extract; ACW: *P. halepensis* Mill. core water extract; BPW: *P. brutia* Ten. petal water extract; BCW: *P. brutia* Ten. core water extract; PPW: *P. pinea* L. petal water extract; PCW: *P. pinea* L. core water extract; IC_50_: half-maximal inhibitory concentration; Vit C: vitamin C as a reference. Each letter on the table represents a significant difference (*p* ≤ 0.05). Results are mean ± SD (n = 3).

**Table 5:** Inhibition of anticancer activity (%).

Extracts	% Inhibition HeLa ^a^	% Inhibition HepG2 ^b^	% Inhibition HEK-293 ^c^
APW	66.32 ± 7.38	57.74 ± 6.22	11.22 ± 2.56
ACW	62.77 ± 3.64	47.22 ± 7.42	14.94 ± 1.55
BPW	58.08 ± 4.05	27.23 ± 6.06	13.45 ± 3.62
BCW	57.51 ± 7.68	24.61 ± 8.01	10.23 ± 4.04
PPW	57.38 ± 3.17	22.37 ± 1.33	8.88 ± 2.45
PCW	47.35 ± 4.82	20.65 ± 3.21	9.12 ± 1.76
Tamoxifen	59.89 ± 4.12	70.33 ± 3.91	68.63 ± 2.46

APW: *P. halepensis* Mill. petal water extract; ACW: *P. halepensis* Mill. core water extract; BPW: *P. brutia* Ten. petal water extract; BCW: *P. brutia* Ten. core water extract; PPW: *P. pinea* L. petal water extract; PCW: *P. pinea* L. core water extract; HeLa cell line: human epithelial cervix carcinoma; HepG2 cell line: hepatic cancer cell line; HEK-293 cell line: human embryonic kidney cell line; tamoxifen at 100 µM: reference. Extracts concentration: 50 µg/mL. Each letter (^a–c^) on the table represents a significant difference (*p* ≤ 0.05). Results are mean ± SD (n = 3).

**Table 6:** Antidiabetic activity (%).

Extracts	α-Glucosidase	
	% Inhibition	IC_50_ (µg/mL)
APW	77.20 ± 1.98 *	24.70 ± 1.34 *
ACW	na	na
BPW	na	na
BCW	na	na
PPW	na	na
PCW	na	na
Acarbose	78.42 ± 0.62	4.98 ± 0.24

APW: *P. halepensis* Mill. petal water extract; ACW: *P. halepensis* Mill. core water extract; BPW: *P. brutia* Ten. petal water extract; BCW: *P. brutia* Ten. core water extract; PPW: *P. pinea* L. petal water extract; PCW: *P. pinea* L. core water extract; na: non-active; acarbose at 50 µg/mL: reference; IC_50_: Half-maximal inhibitory concentration; * significant difference (*p* ≤ 0.05). Results are mean ± SD (n = 3).

**Table 10:** Correlation coefficients R^2^ of total phenolic content, reducing sugar content, antioxidant activity, anticancer activity, and antidiabetic activity.

Variables	TPC	RSC	% Inhibition DPPH	% Inhibition HepG2	% Inhibition HeLa	% Inhibition α-Glucosidase
TPC	1	−0.017	0.942	0.788	0.950	0.917
RSC	−0.017	1	−0.297	0.401	0.067	0.076
% inhibition DPPH	0.942	−0.297	1	0.679	0.921	0.774
% inhibition HepG2	0.788	0.401	0.679	1	0.841	0.618
% inhibition HeLa	0.950	0.067	0.921	0.841	1	0.778
% inhibition α-glucosidase	0.917	0.076	0.774	0.618	0.778	1

TPC: total phenolic content; RSC: reducing sugar content; DPPH: antioxidant activity and HepG2 and HeLa anticancer activity; α-glucosidase: antidiabetic activity.

## Data Availability

Data will be made available on request.
